# Downregulation of HNRNPA1 induced neoantigen generation via regulating alternative splicing

**DOI:** 10.1186/s10020-024-00849-0

**Published:** 2024-06-12

**Authors:** Yaoqi Sun, Bing Xiong, Xueqian Shuai, Jiale Li, Chunyan Wang, Jing Guo, Zhongping Cheng, Shupeng Liu

**Affiliations:** 1 Department of Obstetrics and Gynecology, Shanghai Tenth People’s Hospital, School of Medicine, Tongji University, Shanghai, 200072 China; 2 Anhui University of Science and Technology, Huainan, 232001 China

**Keywords:** Alternative splicing, Cancer immunology, HNRNPA1, Neoantigen

## Abstract

**Background:**

Immunotherapies effectively treat human malignancies, but the low response and resistance are major obstacles. Neoantigen is an emerging target for tumor immunotherapy that can enhance anti-tumor immunity and improve immunotherapy. Aberrant alternative splicing is an important source of neoantigens. HNRNPA1, an RNA splicing factor, was found to be upregulated in the majority of tumors and play an important role in the tumor immunosuppressive microenvironment.

**Methods:**

Whole transcriptome sequencing was performed on shHNRNPA1 SKOV3 cells and transcriptomic data of shHNRNPA1 HepG2, MCF-7M, K562, and B-LL cells were downloaded from the GEO database. Enrichment analysis was performed to elucidate the mechanisms underlying the activation of anti-tumor immunity induced by HNRNPA1 knockdown. mRNA alternative splicing was analyzed and neoantigens were predicted by JCAST v.0.3.5 and Immune epitope database. The immunogenicity of candidate neoantigens was calculated by Class I pMHC Immunogenicity and validated by the IFN-γ ELISpot assay. The effect of shHNRNPA1 on tumor growth and immune cells in vivo was evaluated by xenograft model combined with immunohistochemistry.

**Results:**

HNRNPA1 was upregulated in a majority of malignancies and correlated with immunosuppressive status of the tumor immune microenvironment. Downregulation of HNRNPA1 could induce the activation of immune-related pathways and biological processes. Disruption of HNRNPA1 resulted in aberrant alternative splicing events and generation of immunogenic neoantigens. Downregulation of HNRNPA1 inhibited tumor growth and increased CD8^+^ T cell infiltration in vivo.

**Conclusion:**

Our study demonstrated that targeting HNRNPA1 could produce immunogenic neoantigens that elicit anti-tumor immunity by inducing abnormal mRNA splicing. It suggests that HNRNPA1 may be a potential target for immunotherapy.

**Supplementary Information:**

The online version contains supplementary material available at 10.1186/s10020-024-00849-0.

## Background

Immunotherapies, including immune checkpoint blockade (ICB), adoptive cell therapy (ACT), and oncolytic viruses, have changed the landscape for many malignancies treatment (Kennedy and Salama 2020; Bagchi et al. 2021; Maalej et al. 2023; Hemminki et al. 2020; Saxena et al. 2021). However, therapeutic resistance limits the application of these therapeutic strategies (Bagchi et al. 2021). New immunotherapeutic strategies must be explored to improve response rates and achieve broader efficacy.

Neoantigens characterized by high antigenicity, unique tumor specificity, and avoiding of T-cell central tolerance, displayed the advantage of enhancing immunotherapy response (Hu et al. 2018; Xie et al. 2023). Personalized neoantigen-vaccines were found to prime neoantigen-specific T cells with higher cytotoxicity and tumor infiltration (Awad et al. 2022; Ott et al. 2017). Neoantigen-specific T cells were identified in 67% of treatment-refractory patients in a breast cancer cohort (Zacharakis et al. 2022). And 66.67% of vaccinated patients developed neoantigen-specific immune response and had prolonged progression-free survival time in a microsatellite stability (MSS)-advanced colorectal cancer cohort (Yu et al. 2023). The positive correlation of neoantigen loads with the survival of patients receiving immunotherapy were also reported in a phase I clinical trial of high-grade glioma (HGG) (NCT01470794) and a multi-center phase II trial (NCT02901301) of advanced gastric cancer (Awad et al. 2022; Accomando et al. 2020). In addition, the good tolerance, consistent humoral and cellular immune responses elicited by neoantigen had been proved (Rojas et al. 2023). These findings indicate that tumor-specific neoantigen would be an attractive immunotherapeutic strategy that possess the efficacy and safety in tumor treatment.

RNA splicing is a highly conserved process to generate mature mRNA in eukaryotic cells. Aberrant alternative mRNA splicing in malignant cells is an important source of tumor-specific neoantigens aside from genomic mutations (Shen et al. 2019; Frankiw et al. 2019; Pan et al. 2021). SF3B1 mutation induced generation of immunogenic neoantigens and neoantigen-specific memory CD8^+^ T cells in uveal melanoma (Bigot et al. 2021). Pharmacological disruption of RBM39 also generated tumor-specific neoantigens and induced anti-tumor immunity (Lu et al. 2021). These observations demonstrated the potential of targeting alternative splicing factors to induce neoantigen generation and elicit immune responses.

Heteronuclear ribonucleoprotein A1 (HNRNPA1) is a key RNA-splicing factor that modulates splice site usage, polyadenylation, and cleavage efficiency, which plays important roles in mRNA stability, transport, and metabolism (Hamilton et al. 1997). HNRNPA1 was aberrantly expressed in many malignancies and was reported to promote tumorigenesis and progression through regulating alternative splicing of downstream genes (Roy et al. 2017). It was well known that HNRNPA1 controlled the metabolic switch from oxidative phosphorylation to aerobic glycolysis by regulating the alternative splicing of pyruvate kinase (PK). HNRNPA1 binds to the splice sites flanking exon 9, leading to exon 9 exclusion and exon 10 inclusion, producing pyruvate kinase M2 isoform (PKM2) in most malignant cells, but shifts to downstream intronic sites, leading to 9 inclusion, producing pyruvate kinase M1 isoform (PKM1) in normal cells (Chen et al. 2010, 2012; David et al. 2010). PKM2, instead of PKM1, is usually re-expressed in malignancies and promotes aerobic glycolysis, facilitating the uptake of more energy needed for tumor proliferation (David et al. 2010; Sun et al. 2017; Gu et al. 2017; Zhu et al. 2021; Yan et al. 2021). HRNNPA1 also regulated the splicing of the Myc-interacting partner Max and induced generation of Delta Max, which activated glycolytic genes expression and promoted GBM cell proliferation (Babic et al. 2013). In addition, HNRNPA1 also contributed to enzalutamide resistance of prostate cancer cells via up-regulating the expression of a splicing variant of androgen receptor (AR), AR-V7 (Tummala et al. 2017). It suggested the pivotal role of HNRNPA1 in tumorigenesis. However, whether HNRNPA1 induces neoantigen generation via modulating RNA alternative splicing remains unknown.

Here, we found a positive correlation between HNRNPA1 mRNA expression levels and the tumor immunosuppressive microenvironment by bioinformatics analysis. Whole transcriptome sequencing revealed that the disruption of HNRNPA1 led to abnormal pre-mRNA alternative splicing in tumor cells. Some of the coding transcript variants derived from alternative splicing were found to generate neoantigens binding to HLA-A02:01 and HLA-A03:01. And these neoantigens activated T cell, elicited IFN-γ production in vitro, and displayed the potential immunogenicity. HNRNPA1 downregulation also increased CD8^+^T cell infiltration in tumor tissues and inhibited tumor progression in vivo. Our findings showed that downregulation of HNRNPA1 induced neoantigen generation via disrupting alternative splicing and that HNRNPA1 may serve as a novel immunotherapeutic target in cancer treatment.

## Methods

### Data resources

The RNA sequencing and related clinical data of The Cancer Genome Atlas (TCGA), Therapeutically Applicable Research to Generate Effective Treatments (TARGET), and the Genotype-Tissue Expression (GTEx) were obtained from the UCSC Xena website (http://xena.ucsc.edu/*).* The SRA file and expression matrix of shHNRNPA1 HepG2, MCF-7M, K562, and B-LL (Supplementary Table S1) were downloaded from the Gene Expression Omnibus (GEO) database (https://www.ncbi.nlm.nih.gov/geo/*).*

### Analysis of HNRNPA1 expression in pan-cancer

To generate the normalized expression dataset, samples with an expression level of 0 were filtered out and log2 transformation was performed on each observation. The expression levels of HNRNPA1 in the tumor and corresponding normal tissues were compared using unpaired Student’s t-test in the ggplot2 package (version 3.4.2). Univariate Cox regression analysis was performed to estimate the prognostic value of HNRNPA1 by calculating the hazard ratio (HR) and 95% confidence interval (CI) using the survival (version 3.5) and forestplot (version 3.16) packages. The ESTIMATE package (version 1.0.13) was used to determine the immune score and stromal score (Yoshihara et al. 2013). The CIBERSORT package (version 0.1.0) was applied to assess the levels of 22 infiltrating immune cells (Newman et al. 2015). The GSEAbase package (1.60.1) and GSVA package (version 1.46.0) were used to evaluate the levels of 28 infiltrating immune cells (Barbie et al. 2009). Immune Cell Abundance Identifier (ImmuCellAI) is a tool to estimate the abundance of 24 immune cells from expression dataset. Pan-cancer ImmuCellAI were downloaded from https://guolab.wchscu.cn/ImmuCellAI//#!/ (Miao et al. 2020). The immunophenoscore (IPS) includes four main parts (effector cells (EC), immunosuppressive cells (SC), MHC molecules (MHC), and immunomodulators/checkpoints (CP)) that determine immunogenicity and was calculated on a scale of 0–10 based on the gene expression in representative cell type (Charoentong et al. 2017). Pan-cancer IPSs were downloaded from the Cancer Immune Group Atlas (TCIA, https://tcia.at/home*).* The correlation of HNRNPA1 expression with EstimateScore, immune cell infiltration, and IPS was evaluated across 33 tumors using the Pearson method. A *P-value* < 0.05 was considered as statistically significant.

### HNRNPA1 downregulation by shRNA

SKOV3 (RRID: CVCL_0532), A2780 (RRID: CVCL_0134) and ID8 (RRID: CVCL_VA22) cells were obtained from the American Type Culture Collection (ATCC). SKOV3 and A2780 cells were cultured in RPMI1640 (catalog no. C11875500BT, Gibco) containing 10% FBS (catalog no. FSP500, Excell Bio). ID8 was cultured in DMEM (catalog no. C11995500BT, Gibco) containing 10% FBS. All the cell lines have been tested and authenticated utilizing STR profiling every year. Lentivirus containing shRNA sequence targeting human HNRNPA1 (5’- CAACAATCAGTCTTCAAATTT-3’) or mouse HNRNPA1 (5’-ATGACTCTGTGGATAAGATTG-3’) were purchased from OBiO Inc. (Shanghai, China). SKOV3, A2780 and ID8 cells were infected with lentivirus at an MOI of 20 and selected with 4 µg/mL puromycin (catalog no. ST551-10 mg, Beyotime). The downregulation of HNRNPA1 in tumor cells was validated by qRT-PCR analysis.

### qRT-PCR and whole transcriptome sequencing

Cells were directly lysed with RNA Isolater Total RNA Extraction Reagent (catalog no. R401-01, Vazyme) after PBS washing, and total RNA was extracted according to the manufacturer’s instructions. mRNA concentration was measured using NanoDrop One (Thermo Fisher Scientific). 1–2 µg of mRNA was used for the reverse transcription reaction using the PrimeScript™ RT Master Mix kit (catalog no. RR036A, Takara). TB Green®Premix Ex Taq™II (catalog no. RR820A, Takara) was used for qRT-PCR with ACTB as the internal control. Gene-specific primers for qRT-PCR are listed in Supplementary Table S2. qRT-PCR was performed using the QuantStudio Dx machine (Thermo Fisher Scientific). Data were analyzed by 2^−△△Ct^ method.

For whole transcriptome sequencing of SKOV3 cells, paired-end reads were aligned to the human genome (H. sapiens, GRCh38). Reads with good mapping quality that aligned to genomic exons were counted to generate a table of counts for each gene. Differential gene expression analysis was performed using the R package DESeq2 (version 1.38.3) (Love et al. 2014). Genes with fold change > 1.5 and *P-value* < 0.05 were considered significantly differentially expressed. To find enriched functional annotations for differentially expressed genes (DEGs), Kyoto Encyclopedia of Genes (KEGG) and Gene Ontology (GO) analysis was performed using the clusterProfiler package (version 4.6.2), with *P-value* < 0.05 as the threshold (Yu et al. 2012).

### Analysis of alternative splicing

mRNA alternative splicing was analyzed using RNA-seq data via Multivariate Analysis of Transcript Splicing algorithm (rMATS 4.1.2). The significantly differential alternative splicing (AS) events were identified according to using a cutoff at FDR < 5% and |IncLevelDifference| ≥ 0.01 (Shen et al. 2014).

### Epitope predictions and peptide libraries

Protein isoform identification was performed according to published protocol with some modification (Han et al. 2020). Briefly, JCAST v.0.3.5 was applied to generate protein isoforms derived from alternative spliced mRNA transcript variants using reference genome GRCh38.109. And the protein isoforms were sent to the Immune epitope database (IEDB) for antigen prediction using Next-Generation IEDB Tools (MHC-I Binding Predictions, NetMHCpan EL4.1). The predicted epitopes binding to HLA-A*02:01 and HLA-A*03:01 with a percentile rank < 0.1 were selected for the following immunogenicity scoring using Class I pMHC Immunogenicity.

### IFN-γ enzyme-linked immunospots (ELISpot) assay

Peptides were synthesized and HPLC purified by Genscript Inc. (Nanjing, Jiangsu, China). The detailed sequences of the peptides were shown in Table 3. Cryopreserved peripheral blood mononuclear cells (PBMCs) were purchased from OriBiotech and rested in R10 medium (90% RPMI1640 + 10% fetal bovine serum (catalog no. 10,099,141 C, Gibco)). IFN-γ ELISpot assay was performed with ELISpot Plus: Human IFN-γ(ALP) kit (catalog no. 3420-4AST-2, MabTech). In brief, the plate was washed three times with PBS, and incubated with 200µL R10 medium per well at room temperature for 1 h before discarding the medium. The stimulations were added 50µL per well at optimal concentrations: 10 µg/mL peptides, anti-CD3α mAb (1:500) as positive control, and DMSO as negative control. 50µL cell suspension was then added to each well at a density of 2.5 × 10^5^ cells per well for peptides and 10^5^ cells per well for positive control. The plate was incubated at 37℃ for 27 h and then washed 5 times with PBS. The detection antibody, 7-B6-ALP was diluted 1:1000 in PBS (0.5%FBS), 100µL per well. After incubation at room temperature for 2 h, the plate was washed 5 times with PBS. 100µL Streptavidin-ALP (1:1000 in PBS (0.5%FBS)) was added into each well and incubated at room temperature for 1 h. 100µL BCIP/NBT-plus was then added into each well after washing the plate 5 times with PBS. When distinct spots emerged, the color development was stopped by washing extensively in tap water, to remove the plate frame from the plastic tray and rinse the underside of the membranes. After drying the plate, an automatic plate reader with appropriate parameters set beforehand was used to read the plate.

### Xenograft model

Five weeks old C57BL/6J mice were purchased from Shanghai Laboratory Animal Center (SLAC, China) and acclimated for 1 week before experiments. A total of 50µL cell suspension (5 × 10^6^ ID8 ovarian cancer cells transfected with shHNRNPA1 or shNC virus) were subcutaneously injected into the upper flank region of each mouse with 50µL Matrigel (catalog no.356,234, Corning). Tumor volume was measured every three days by vernier caliper, and calculated based on length × width^2^/2. At the end of experiment, mice were sacrificed, and subcutaneous tumors were harvested for immunohistochemistry analysis. The percentage of DAB positve cells were analyzed using ImageJ. All animal-related experiments were acknowledged by the Experimental Animal Ethics Committee of Shanghai Tenth People’s Hospital, School of Medicine, Tongji University (SHDSYY-2023-Y3425).

## Results

### The HNRNPA1 expression levels correlate with immunosuppressive status of TIME across pan-cancer

**Fig. 1:**
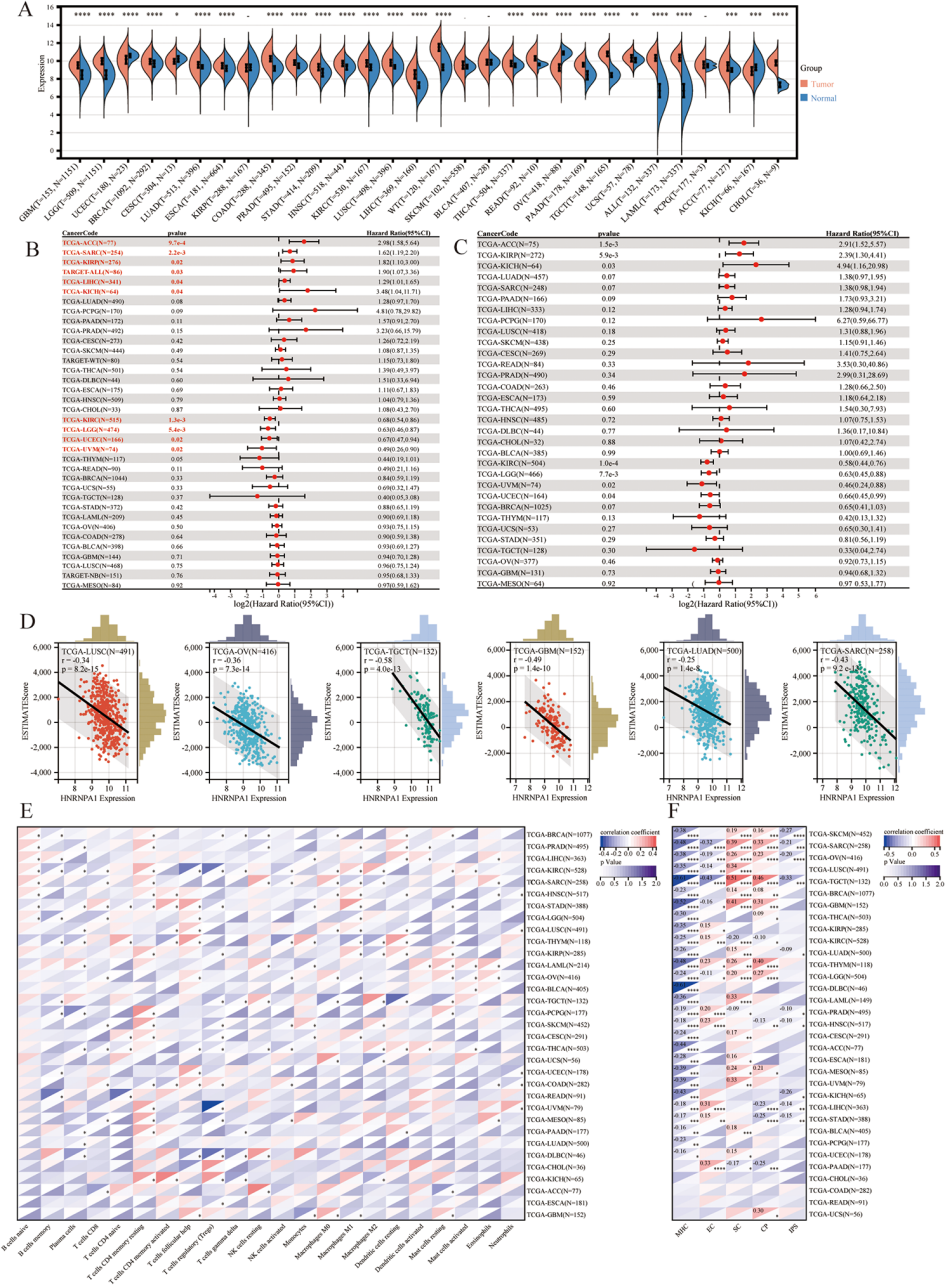
Pan-cancer analysis of HNRNPA1. (**A**) Comparison of HNRNPA1 expression levels between tumors and normal tissues. (**B**) Univariate cox regression results of HNRNPA1 for OS from TCGA and TARGET databases. (**C**) Univariate cox regression results of HNRNPA1 for DSS from TCGA database. (**D**) Association between HNRNPA1 expression and EstimateScore in LUSC, NB, OV, SARC, KIRC, and TGCT. (**E**) Correlation between HNRNPA1 expression and the proportion of 22 immune infiltrating cells. (**F**) Correlation between HNRNPA1 expression and immunophenoscore

Before analyzing the involvement of HNRNPA1 in neoantigen generation, we intended to investigate the correlation of HNRNPA1 expression with tumor immune microenvironment (TIME) firstly due to the crucial roles of neoantigen in tumor immune response. The expression of HNRNPA1 across pan-cancer was evaluated using data from TCGA, TARGET, and GTEx datasets. Compared with normal tissues, HNRNPA1 was significantly upregulated in 22 of 30 cancer types, including brain lower-grade glioma (LGG), acute myeloid leukemia (LAML), colon adenocarcinoma (COAD), and so on (*p* < 0.05), while it was downregulated in kidney chromophobe (KICH) and three gynecological carcinomas, including cervical squamous cell carcinoma and endocervical adenocarcinoma (CESC), uterine corpus endometrial carcinoma (UCEC) and ovarian serous cystadenocarcinoma (OV) (*p* < 0.05) (Fig. 1A). No differences were found in kidney renal papillary cell carcinoma (KIRP), skin cutaneous melanoma (SKCM), bladder urothelial carcinoma (BLCA), and pheochromocytoma and paraganglioma (PCPG) (Fig. 1A). These results indicated that HNRNPA1 was abnormally expressed across multiple malignancies. The clinical relevance of HNRNPA1 expression in different malignancies was estimated using univariate Cox regression analysis. The results showed that HNRNPA1 expression was a risk factor for overall survival (OS) in adrenocortical carcinoma (ACC), sarcoma (SARC), KIRP, acute lymphoblastic leukemia (ALL), liver hepatocellular carcinoma (LIHC), and KICH, but a protector in kidney renal clear cell carcinoma (KIRC), LGG, UCEC, and uveal melanoma (UVM) (Fig. 1B). A similar analysis was also performed against disease-specific survival (DSS) to avoid deviation of people who did not die from specific cancer. The similar results were observed in most tumors except for SARC, ALL, and LIHC (Fig. 1C). It revealed that HNRNPA1 expression was associated with the prognosis of diverse malignancies. To further elucidate the correlation of HNRNPA1 with the TIME, the immune infiltration analyses were performed across pan-cancer. The EstimateScore, which integrates ImmuneScore and StromalScore, was calculated to evaluate the relationship between HNRNPA1 expression and immune infiltration. HNRNPA1 expression was negatively correlated with the EstimateScore in 24 of 33 cancers, and positively correlated with EstimateScore in KIRC (Fig. 1D; Table 1). Then, the CIBERSORT, ssGSEA and ImmuCellAI algorithms were implemented to further investigate the potential association between HNRNPA1 expression and infiltration levels of specific immune cells. Varied correlations were observed across immune cells and tumors (Fig. 1E, Supplementary Fig. 1A-B). Consistently, HNRNPA1 levels were positively correlated with several immunosuppressive cells and naïve cells, such as Type 2 T helper cell, nTreg, iTreg, CD4 naïve, CD8 naïve and B cells naïve, while negatively correlated with activated CD8 T cell, γδ T cells and activated NK cells in several malignancies (Fig. 1E, Supplementary Fig. 1A-B). Subsequent IPS analysis was performed to evaluate the immunogenicity of tumors with different HNRNPA1 expression level. The results showed a positive correlation between HNRNPA1 expression and SC modules or CP modules in most tumors (Fig. 1F). It was worth mentioning that HNRNPA1 expression was negatively correlated with MHC modules in all tumors, while 28 of 33 were statistically significant (Fig. 1F). It suggested that higher expression of HNRNPA1 may be related to the downregulation of MHC molecules, which could avoid recognition by T cells and promote immune escape. Additionally, IPS was also a superior predictor of response to anti-cytotoxic T lymphocyte antigen-4 (CTLA-4) and anti-programmed cell death protein 1 (anti-PD-1) (Charoentong et al. 2017). The correlation indicated that higher expression of HNRNPA1 might present with a worse response for checkpoint inhibitor-based immunotherapy in 10 types of malignancies (Fig. 1F; Table 2). In brief, these pan-cancer analyses revealed the negative correlation of HNRNPA1 with TIME in most malignancies, suggesting that HNRNPA1 targeting may help remodel the TIME and activate anti-tumor immunity.

**Table 1:** Correlation of HNRNPA1 expression with StromalScore, ImmuneScore, and EstimateScore across pan-cancer

Cancer type	StromalScore		ImmuneScore		ESTIMATEScore	
	*R*	*P* value	*R*	*P* value	*R*	*P* value
TCGA-GBM(*N* = 152)	-0.42	9.00E-08	-0.51	1.24E-11	-0.49	1.36E-10
TCGA-LGG(*N* = 504)	-0.38	5.81E-19	-0.22	9.97E-07	-0.29	4.72E-11
TCGA-CESC(*N* = 291)	-0.05	4.14E-01	-0.26	1.03E-05	-0.19	1.22E-03
TCGA-LUAD(*N* = 500)	-0.23	2.33E-07	-0.24	1.06E-07	-0.25	1.42E-08
TCGA-COAD(*N* = 282)	-0.15	1.16E-02	-0.14	2.04E-02	-0.15	1.02E-02
TCGA-LAML(*N* = 149)	-0.27	1.09E-03	-0.33	3.06E-05	-0.33	5.15E-05
TCGA-BRCA(*N* = 1077)	-0.08	1.15E-02	-0.17	3.81E-08	-0.14	2.40E-06
TCGA-ESCA(*N* = 181)	-0.12	1.07E-01	-0.20	5.79E-03	-0.18	1.73E-02
TCGA-SARC(*N* = 258)	-0.30	7.88E-07	-0.46	1.05E-14	-0.43	9.17E-13
TCGA-KIRP(*N* = 285)	-0.01	8.23E-01	-0.17	3.56E-03	-0.12	5.17E-02
TCGA-STAD(*N* = 388)	-0.19	1.13E-04	-0.10	3.90E-02	-0.16	1.27E-03
TCGA-PRAD(*N* = 495)	-0.07	1.21E-01	-0.09	3.67E-02	-0.09	4.41E-02
TCGA-UCEC(*N* = 178)	-0.27	2.13E-04	-0.30	4.06E-05	-0.32	1.39E-05
TCGA-HNSC(*N* = 517)	-0.12	7.65E-03	-0.06	1.90E-01	-0.10	2.79E-02
TCGA-KIRC(*N* = 528)	0.28	1.23E-10	-0.05	2.27E-01	0.10	2.39E-02
TCGA-LUSC(*N* = 491)	-0.31	4.74E-12	-0.34	2.15E-14	-0.34	8.25E-15
TCGA-THYM(*N* = 118)	-0.16	9.36E-02	-0.04	6.31E-01	-0.11	2.22E-01
TCGA-LIHC(*N* = 363)	-0.10	6.47E-02	-0.06	2.91E-01	-0.08	1.30E-01
TCGA-THCA(*N* = 503)	-0.02	6.11E-01	-0.17	1.02E-04	-0.12	5.75E-03
TCGA-MESO(*N* = 85)	0.01	9.24E-01	-0.30	4.83E-03	-0.20	6.05E-02
TCGA-READ(*N* = 91)	-0.10	3.61E-01	-0.13	2.07E-01	-0.12	2.60E-01
TCGA-SKCM(*N* = 452)	-0.15	1.74E-03	-0.26	1.35E-08	-0.24	3.60E-07
TCGA-PAAD(*N* = 177)	0.14	5.77E-02	0.10	1.75E-01	0.13	8.72E-02
TCGA-OV(*N* = 416)	-0.25	1.43E-07	-0.39	3.17E-16	-0.36	7.29E-14
TCGA-TGCT(*N* = 132)	-0.35	3.67E-05	-0.54	3.04E-11	-0.58	3.96E-13
TCGA-PCPG(*N* = 177)	-0.01	8.73E-01	-0.25	9.54E-04	-0.13	7.40E-02
TCGA-UVM(*N* = 79)	-0.48	9.52E-06	-0.46	2.16E-05	-0.49	5.32E-06
TCGA-UCS(*N* = 56)	-0.32	1.58E-02	-0.28	3.75E-02	-0.34	1.08E-02
TCGA-BLCA(*N* = 405)	-0.28	1.24E-08	-0.23	1.93E-06	-0.27	2.29E-08
TCGA-ACC(*N* = 77)	-0.21	7.09E-02	-0.35	1.62E-03	-0.30	7.10E-03
TCGA-KICH(*N* = 65)	-0.03	8.37E-01	-0.09	4.73E-01	-0.06	6.07E-01
TCGA-CHOL(*N* = 36)	0.11	5.16E-01	0.10	5.48E-01	0.11	5.13E-01
TCGA-DLBC(*N* = 46)	-0.02	9.21E-01	-0.15	3.14E-01	-0.10	4.97E-01

**Table 2:** Correlation of HNRNPA1 expression with IPS score across pan-cancer

Cancer type	MHC		EC		SC		CP		IPS	
	*R*	*P* value	*R*	*P* value	*R*	*P* value	*R*	*P* value	*R*	*P* value
TCGA-GBM(*N* = 152)	-0.52	8.93E-12	-0.16	4.71E-02	0.41	1.10E-07	0.31	1.08E-04	-0.02	8.21E-01
TCGA-LGG(*N* = 504)	-0.24	5.77E-08	-0.11	1.74E-02	0.20	5.65E-06	0.27	3.47E-10	-0.07	9.88E-02
TCGA-CESC(*N* = 291)	-0.24	4.37E-05	-0.05	4.32E-01	0.17	3.34E-03	0.11	7.29E-02	-0.03	5.64E-01
TCGA-LUAD(*N* = 500)	-0.26	6.64E-09	-0.02	7.11E-01	0.15	8.50E-04	0.05	2.89E-01	-0.09	3.52E-02
TCGA-COAD(*N* = 282)	-0.07	2.54E-01	0.05	4.29E-01	0.05	4.10E-01	0.03	6.08E-01	0.07	2.30E-01
TCGA-LAML(*N* = 149)	-0.36	8.58E-06	-0.11	1.79E-01	0.33	4.12E-05	0.15	7.45E-02	-0.02	8.11E-01
TCGA-BRCA(*N* = 1077)	-0.23	4.22E-14	0.02	5.01E-01	0.14	3.37E-06	0.08	5.75E-03	0.02	5.70E-01
TCGA-ESCA(*N* = 181)	-0.28	1.69E-04	-0.01	9.34E-01	0.16	2.75E-02	0.00	9.99E-01	-0.11	1.31E-01
TCGA-SARC(*N* = 258)	-0.48	3.17E-16	-0.32	1.54E-07	0.39	8.92E-11	0.33	8.11E-08	-0.21	8.08E-04
TCGA-KIRP(*N* = 285)	-0.35	1.75E-09	0.15	1.20E-02	-0.02	7.39E-01	0.03	6.13E-01	-0.06	3.07E-01
TCGA-STAD(*N* = 388)	-0.17	8.01E-04	0.15	2.29E-03	0.06	2.46E-01	-0.25	9.12E-07	-0.15	2.34E-03
TCGA-PRAD(*N* = 495)	-0.19	1.49E-05	0.20	8.50E-06	-0.09	3.74E-02	-0.05	2.85E-01	-0.10	2.19E-02
TCGA-UCEC(*N* = 178)	-0.16	2.87E-02	0.10	1.96E-01	0.15	4.43E-02	0.04	6.22E-01	-0.05	4.96E-01
TCGA-HNSC(*N* = 517)	-0.18	4.04E-05	0.23	9.60E-08	0.07	1.09E-01	-0.13	3.97E-03	-0.10	2.77E-02
TCGA-KIRC(*N* = 528)	-0.25	4.34E-09	0.15	4.71E-04	-0.20	2.55E-06	-0.10	1.88E-02	-0.05	2.72E-01
TCGA-LUSC(*N* = 491)	-0.35	2.78E-15	-0.14	1.79E-03	0.34	2.38E-14	0.09	5.65E-02	-0.07	1.00E-01
TCGA-THYM(*N* = 118)	-0.48	4.45E-08	0.23	1.33E-02	0.26	4.34E-03	0.40	8.21E-06	0.10	2.75E-01
TCGA-LIHC(*N* = 363)	-0.18	4.59E-04	0.31	1.40E-09	-0.10	6.22E-02	-0.23	1.05E-05	-0.14	6.83E-03
TCGA-THCA(*N* = 503)	-0.30	1.12E-11	0.02	6.62E-01	-0.01	8.67E-01	0.09	3.64E-02	-0.07	1.41E-01
TCGA-MESO(*N* = 85)	-0.39	2.07E-04	-0.10	3.42E-01	0.24	2.66E-02	0.21	4.87E-02	-0.06	5.87E-01
TCGA-READ(*N* = 91)	-0.12	2.63E-01	0.15	1.59E-01	-0.05	6.20E-01	-0.16	1.31E-01	-0.08	4.53E-01
TCGA-SKCM(*N* = 452)	-0.38	7.21E-17	-0.07	1.31E-01	0.19	4.37E-05	0.16	7.28E-04	-0.27	6.98E-09
TCGA-PAAD(*N* = 177)	-0.13	8.07E-02	0.33	1.00E-05	-0.17	2.62E-02	-0.25	9.12E-04	-0.01	8.86E-01
TCGA-OV(*N* = 416)	-0.38	1.98E-15	-0.19	1.33E-04	0.26	1.04E-07	0.23	1.35E-06	-0.20	2.67E-05
TCGA-TGCT(*N* = 132)	-0.61	7.54E-15	-0.43	3.32E-07	0.51	3.77E-10	0.46	3.82E-08	-0.33	1.22E-04
TCGA-PCPG(*N* = 177)	-0.23	1.89E-03	-0.03	7.34E-01	0.03	7.41E-01	0.08	2.63E-01	0.02	8.09E-01
TCGA-UVM(*N* = 79)	-0.39	3.62E-04	-0.08	4.70E-01	0.33	2.70E-03	0.19	9.89E-02	-0.12	2.74E-01
TCGA-UCS(*N* = 56)	-0.14	3.13E-01	-0.21	1.28E-01	0.20	1.38E-01	0.30	2.35E-02	0.11	4.18E-01
TCGA-BLCA(*N* = 405)	-0.16	1.53E-03	-0.01	7.79E-01	0.18	3.16E-04	0.03	4.90E-01	-0.09	7.73E-02
TCGA-ACC(*N* = 77)	-0.44	6.41E-05	-0.02	8.68E-01	0.15	2.04E-01	0.03	7.70E-01	-0.21	6.63E-02
TCGA-KICH(*N* = 65)	-0.43	3.64E-04	0.24	5.79E-02	-0.08	5.12E-01	-0.14	2.73E-01	-0.26	3.50E-02
TCGA-CHOL(*N* = 36)	-0.28	1.04E-01	0.15	3.73E-01	-0.18	2.88E-01	-0.19	2.75E-01	-0.03	8.84E-01
TCGA-DLBC(*N* = 46)	-0.61	5.85E-06	0.10	5.17E-01	0.08	5.97E-01	0.01	9.44E-01	-0.22	1.49E-01

### Identification of immune-related signaling pathways in HNRNPA1 knockdown cells

**Fig. 2:**
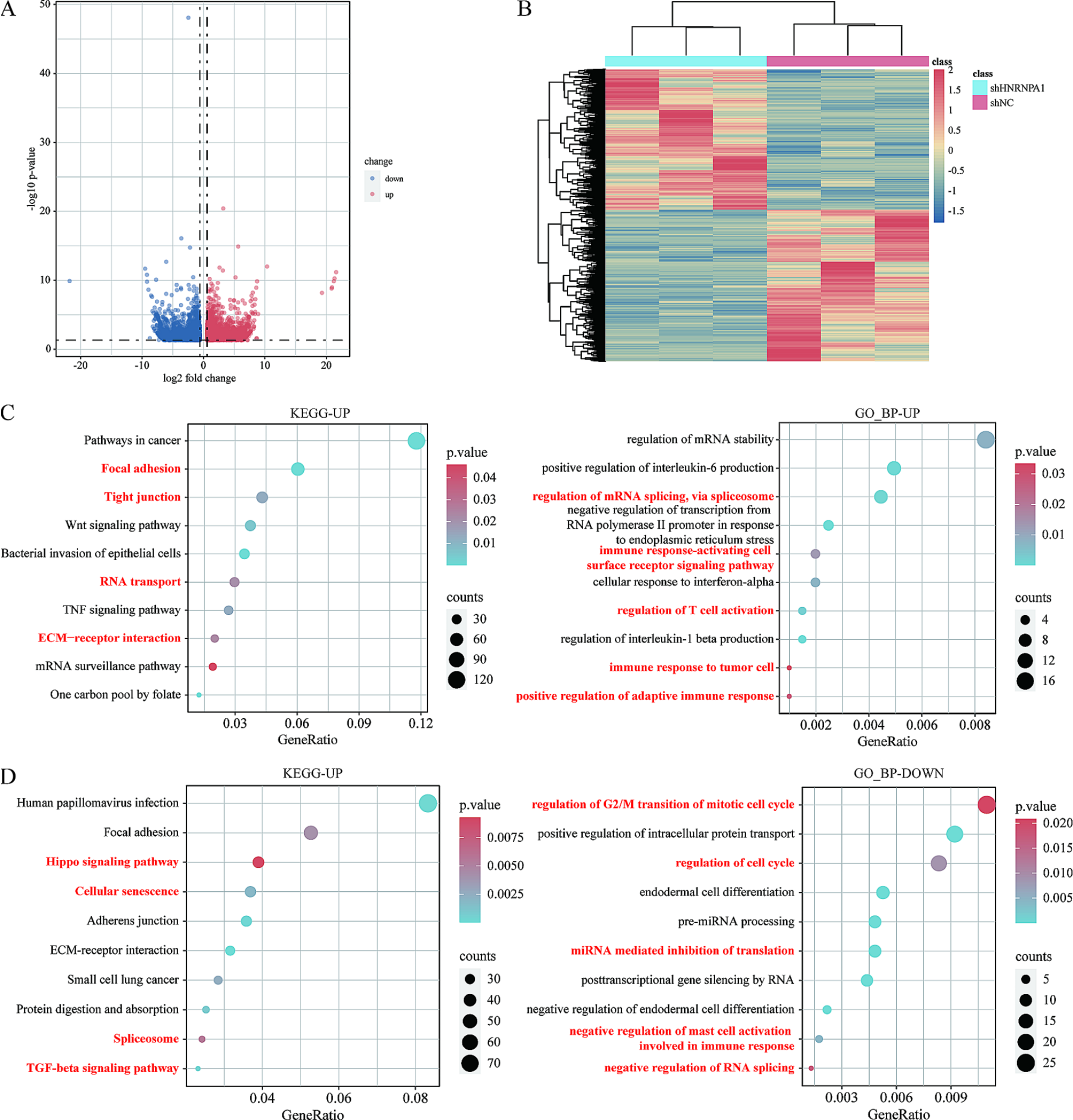
Identification and Functional Enrichment Analysis of DEGs in shHNRNPA1 SKOV3 cells. (**A**) The volcano plot of the differentially expressed genes in shHNRNPA1 SKOV3 cells. The threshold is fold change > 1.5 and *P value* < 0.05. The up-regulated genes are shown in red, while the down-regulated genes are shown in blue. (**B**) Heatmap of a total of 5348 DEGs (*n* = 3). (**C**) Enriched KEGG and GO terms based on up-regulated DEGs (*P value* < 0.05). (**D**) Enriched KEGG and GO terms based on downregulated DEGs (*P value* < 0.05)

To investigate whether targeting HNRNPA1 can activate anti-tumor immunity, we performed whole-transcriptome sequencing on HNRNPA1-knockdown SKOV3 cells, and obtained four published transcriptomic data of different tumor cell lines (MCF-7M, HepG2, K562, and B-LL) from the GEO database. First, the differential expression analysis was performed to identify the specific gene expression profiles of shHNRNPA1 cells. Compared to shNC cells, 2572 upregulated and 2776 downregulated genes were identified in shHNRNPA1 SKOV3 cells, 162 upregulated and 51 downregulated genes in shHNRNPA1 MCF-7M, 1710 upregulated and 2066 downregulated genes in shHNRNPA1 HepG2, 2388 upregulated and 1528 downregulated genes in shHNRNPA1 K562, 10 upregulated and 23 downregulated genes in shHNRNPA1 B-LL (Fig. 2A-B, Supplementary Fig. 2). KEGG and GO analysis were then performed to investigate the signaling pathways and BPs activated in shHNRNPA1 cells. Several KEGG pathways or GO BPs involved in RNA splicing, such as spliceosome in SKOV3, U2-type prespliceosome assembly in HepG2, regulation of mRNA splicing, via spliceosome in MCF-7M, and ncRNA processing in K562, were found to be altered in shHNRNPA1 cells, which may be related to the inherent function of HNRNPA1 as an alternative splicing factor (Fig. 2D, Supplementary Fig. 3A-C). In shHNRNPA1 SKOV3 cells, KEGG analysis showed that upregulated DEGs were mainly enriched in cell adhesion pathways, such as focal adhesion, tight junction, and ECM-receptor interaction, while downregulated DEGs were mainly enriched in Hippo signaling pathway, cellular senescence, and TGF-beta signaling pathway. GO analysis revealed that these upregulated DEGs were mainly enriched in immune-related BPs, including regulation of T cell activation, immune response to tumor cells, etc., and downregulated DEGs were enriched in BPs involved in cell cycle and RNA splicing (Fig. 2C-D). In shHNRNPA1 HepG2, MCF-7M, and B-LL cells, upregulated genes were similarly enriched in several immune-related pathways and BPs, such as interferon-gamma-mediated signaling pathway, antigen processing and presentation, cytokine-mediated signaling pathway, and chemokine-cytokine receptor interaction, indicating the activation of anti-tumor immunity (Supplementary Fig. 3A-B, D). Several pathways and BPs related to cell proliferation, including cell cycle, DNA replication, cell cycle G2/M phase transition, etc., were also downregulated in shHNRNPA1 HepG2, MCF-7M cells (Supplementary Fig. 3A-B). In shHNRNPA1 K562 cells, the upregulated genes were mainly enriched in pathways and BPs related to tumor progression and RNA processing, such as PI3K-Atk signaling pathway, VEGF signaling pathway, ncRNA processing, rRNA processing, regulation of miRNA metabolic process, etc. (Supplementary Fig. 3C). Functional enrichment analysis revealed potential activation of immune-related pathways in shHNRNPA1 cells, suggesting that targeting HNRNPA1 may prime immune responses.

### Downregulation of HNRNPA1 induced aberrantly alternative splicing

**Fig. 3:**
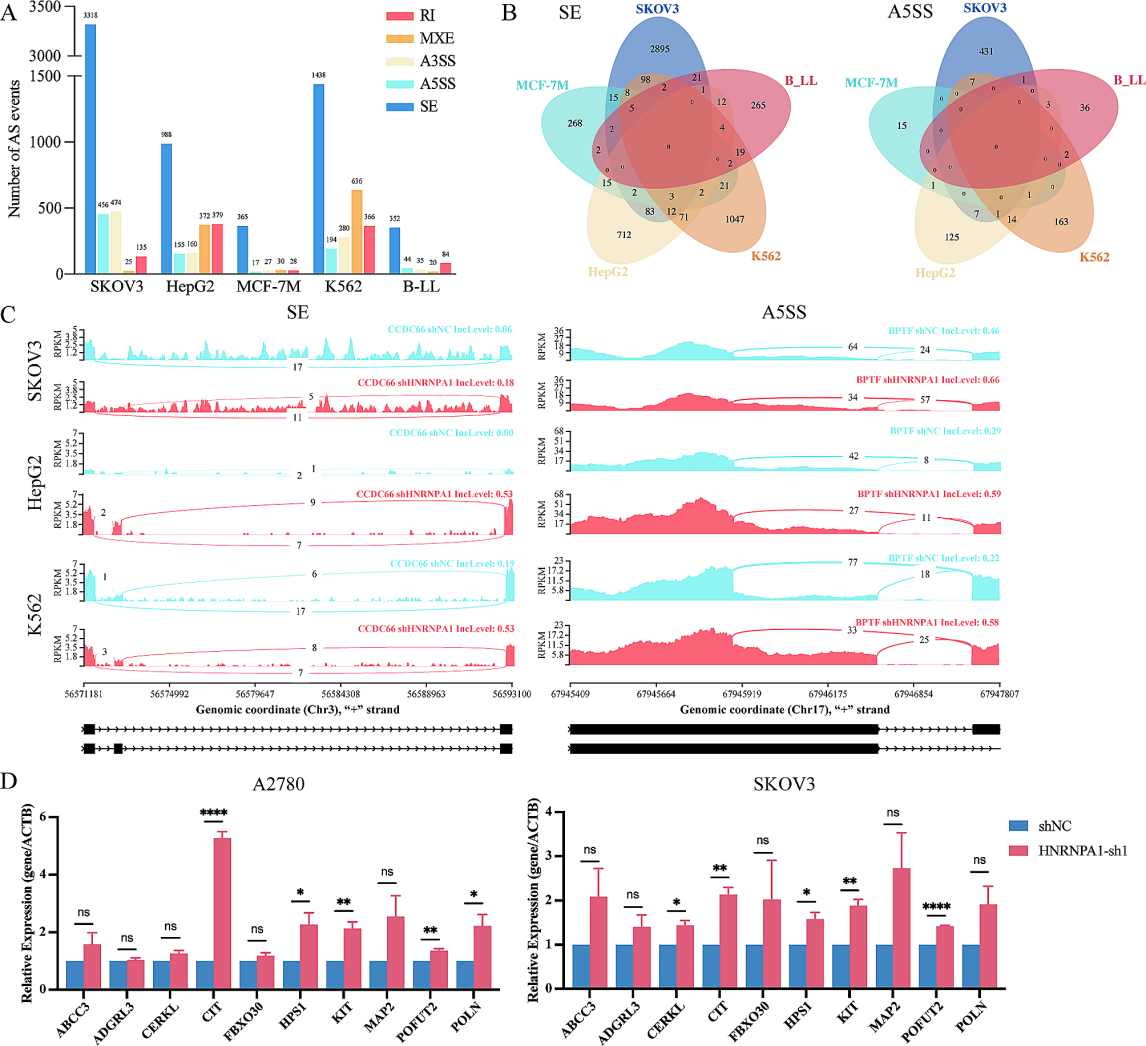
Downregulation of HNRNPA1 induced aberrant alternative splicing. (**A**)The number of 5 basic types of dysregulated alternative splicing events in five tumor cell lines. The threshold is FDR < = 0.05 and |IncLevelDifference|>0.01. (**B**) The Venn diagram of alternative splicing events, SE and A5SS, shared by five tumor cell lines. (**C**) Sashimi plot illustrating the shared skipped exon (left) and 5’splice site (right) induced by HNRNPA1 downregulation in SKOV3, HepG2, and K562. (**D**) qRT-PCR analysis of 10 transcripts in both A2780 and SKOV3 cells infected with shHNRNPA1 lentivirus. Error bars, mean ± SD. (*P < 0.05, **P < 0.01, ***P < 0.001, ****P < 0.0001)

To investigate the regulation of pre-mRNA alternative splicing by HNRNPA1, the global mRNA splicing in cancer cells with or without HNRNPA1 downregulation was analyzed. mRNA expression data from RNA-seq analysis of 5 different tumor cell lines (SKOV3, HepG2, MCF-7M, K562 and B-LL) were included for differential alternative splicing events (ASEs) analysis. It showed that all five basic AS patterns including skipped exon (SE), 5’splice site (A5SS), alternative 3’splice site (A3SS), mutually exclusive exons (MXE) and retained intron (RI) were found to be dysregulated in HNRNPA1 downregulating tumor cells (shHNRNPA1 vs. shNC) of each cell line (Fig. 3A, and Supplementary Fig. 4A). Among these AS patterns, SE was shown to have the highest number of differential ASEs. The number of differential SE ASEs was about 10-fold higher than that of the other four AS patterns (Fig. 3A). The number of ASEs induced by HNRNPA1 downregulation also varied among tumor cell lines, with the highest in SKOV3 (4408 in total) and the lowest in MCF-7M (467 in total) (Supplementary Fig. 4B). There were 3318 SE, 456 A5SS, 474 A3SS, 25 MXE, 135 RI differential ASEs in SKOV3, while 365 SE, 17 A5SS, 27 A3SS, 30 MXE, 28 RI differential ASEs were found in MCF-7M (Fig. 3A). These identified ASEs in tumor cells indicated that HNRNPA1 downregulation induced aberrantly AS of pre-mRNAs. The following intersection analysis showed that no ASEs were found consistently across all 5 cell lines, and no more than 8 SE ASEs were found consistently in 4 cell lines (Fig. 3B, and Supplementary Fig. 5). The number of concurrent ASE among 3 cell lines was higher for each AS patterns than that among 4 cell lines (35 SE, 2 A5SS, 4 A3SS, 2 MXE, and 5 RI), but the percentage remained low (0.63% SE, 0.25% A5SS, 0.46% A3SS, 0.21% MXE, and 0.58% RI) (Supplementary Fig. 5). The examples of concurrent SE and A5SS ASEs in SKOV3, HepG2, and K562 were shown (Fig. 3C). For further verification, 10 transcripts derived from ASEs in SKOV3 with HNRNPA1 downregulation were selected randomly for qRT-PCR analysis using specific primers. It showed that 5 transcripts were up-regulated by downregulation of HNRNPA1 in SKOV3 and A2780, which was consistent with those identified by RNA-seq (Fig. 3D). These data indicated that HNRNPA1 regulated global mRNA splicing in cancer cells and its downregulation induced aberrant AS of mRNA and altered expression of transcript variants of genes.

### Identification of neo-antigen derived from AS

**Fig. 4:**
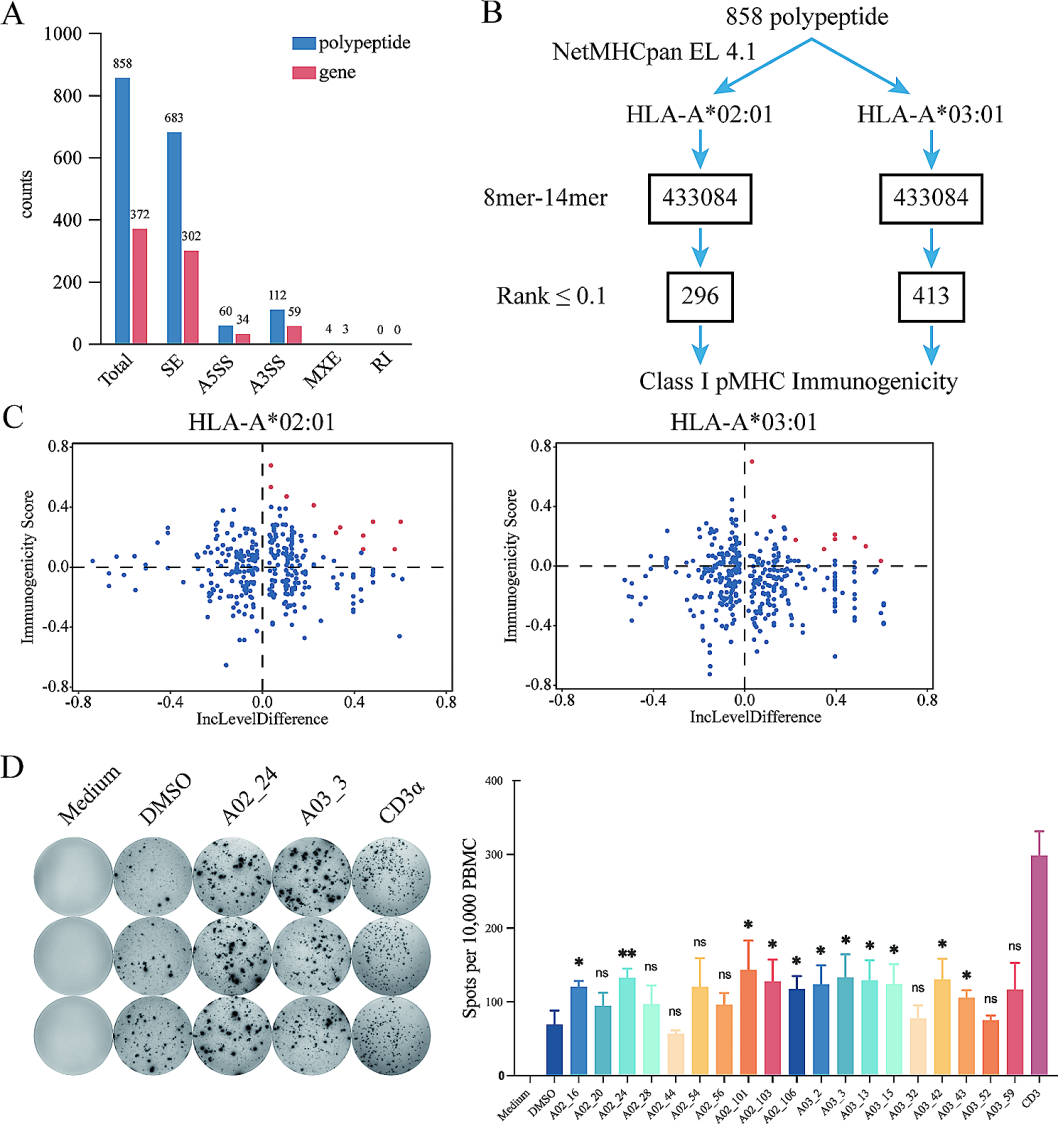
Downregulation of HNRNPA1 induced the production of neoantigens generated by alternative splicing. (**A**) Number of polypeptides and referring genes from AS-derived transcript variants. (**B**) The flowchart of neoantigens identification. (**C**) The dot plot of the immunogenicity score generated by the Class I pMHC Immunogenicity algorithm. The neoantigens selected for further verification are shown in red. (**D**) PBMC were cultured with neoantigens (5µg/mL) and the T cell reactivity was detected by IFN-γ ELISpot assay. Columns represent the mean of triplicate of IFN-γ spots counts for 10^5^ cells, *n* = 3. Error bars, mean ± SD. (*P < 0.05, **P < 0.01, ***P < 0.001, ****P < 0.0001)

AS has been reported to produce neoantigens (Shen et al. 2019; Frankiw et al. 2019; Pan et al. 2021). To investigate neoantigens induced by HNRNPA1 downregulation in tumor cells, transcript variants derived from AS in SKOV3 were translated into polypeptides using JCAST. A total of 858 polypeptides referring to 372 genes were produced from these transcript variants (Fig. 4A). Among these polypeptides, those derived from SE accounted for the highest percentage (683 SE, 60 A5SS, 112 A3SS, and 4 MXE), while no polypeptides were derived from RI (Fig. 4A). All these AS-derived polypeptides were then sent to the NetMHCPan algorithm for neoantigen identification. 296 and 413 neoantigens with rank higher than the threshold (0.1) were found to potentially bind to MHC I allele HLA-A*02:01 and HLA-A*03:01 respectively (Fig. 4B). For further verification, the Class I pMHC Immunogenicity algorithm was used to predict the immunogenicity of these neoantigens. It showed that some neoantigens received immunogenicity score higher than 0, suggesting that the immune response could be elicited by the peptide/allele complex (Fig. 4C). For ease of use, the IncLevelDifference of the transcripts from which neoantigens arising was included for neoantigen selection. Neoantigens (10 for HLA-A*02:01, 9 for HLA-A*03:01) with higher immunogenicity score and higher IncLevelDifference were selected for subsequent functional assays (Fig. 4C; Table 3). To assess immunogenicity in vitro, PBMC of healthy human with MHC I allele HLA-A*02:01 and HLA-A*03:01 were stimulated with neoantigens (Table 3), and then sent for IFN-γ secretion analysis using IFN-γ ELISpot assay. Most of the neoantigens (11/19) selected for evaluation stimulated PBMC to secrete IFN-γ (Fig. 4D, Supplementary Fig. 6). 2 neoantigens (A02-24 and A02-101) binding to HLA-A*02:01 were found to induce a much higher level of IFN-γ than the negative control (Fig. 4D, Supplementary Fig. 6). It indicated that AS-derived neoantigens induced by HNRNPA1 downregulation were recognized by PBMC and displayed immunogenicity.

**Table 3:** The detailed information of selected neoepitopes for IFN-γ ELISpots assay

Code	Peptide	HLA-A allele	Gene	Junction type	Immunogenicity score	Rank	IncLevel difference
A02_16	ALSAWTYFL	02:01	TRIML2	A3SS	0.30319	0.09	0.601
A02_20	ALTGGFHPV	02:01	UNC5B	SE	0.21072	0.07	0.438
A02_24	ALYAGLVVA	02:01	UNC5B	SE	0.11969	0.08	0.438
A02_28	AQLWEDEWEV	02:01	GDPD5	SE	0.67808	0.04	0.037
A02_44	GLPEAGWEL	02:01	SURF4	A3SS	0.41259	0.08	0.223
A02_54	GVAEIPVYI	02:01	POLN	SE	0.26529	0.08	0.338
A02_56	HLYEEEIGAV	02:01	FBXO30	SE	0.47129	0.09	0.105
A02_101	QLWEDEWEV	02:01	GDPD5	SE	0.53394	0.01	0.037
A02_103	QLWEHFQSL	02:01	HPS1	SE	0.11993	0.01	0.575
A02_106	RLDGAINRV	02:01	PHKA2	SE	0.22946	0.03	0.32
A03_2	ALQFLRVTK	03:01	SURF4	A3SS	0.17564	0.06	0.223
A03_3	ALTLPGLLWK	03:01	POFUT2	A3SS	0.13326	0.05	0.53
A03_13	GVSYVVPTK	03:01	KIT	A3SS	0.03496	0.04	0.596
A03_15	ILNPPFEGK	03:01	MAP4	SE	0.19044	0.03	0.481
A03_32	KTPAIPTPK	03:01	NACA	A5SS	0.18121	0.06	0.395
A03_42	RITEEFLGK	03:01	VCAN	SE	0.33149	0.05	0.128
A03_43	RLFFWWFTK	03:01	ABCC3	SE	0.70149	0.08	0.032
A03_52	RTPHTPGTPK	03:01	MAP2	SE	0.11413	0.03	0.347
A03_59	SLGEPLPIGK	03:01	NACA	A5SS	0.21183	0.09	0.395

### Downregulation of HNRNPA1 increased infiltration of T cells in tumor tissues

**Fig. 5:**
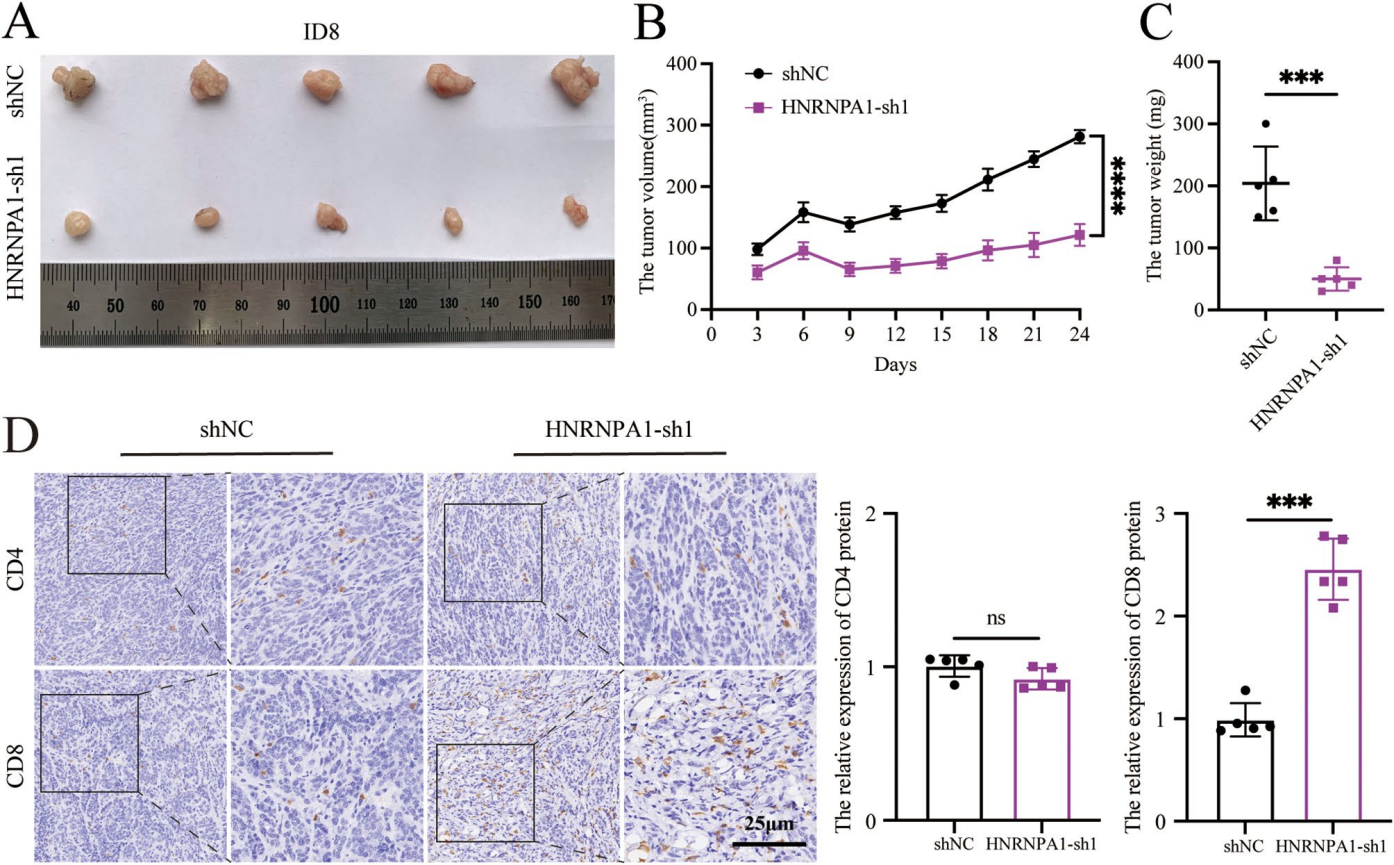
Downregulation of HNRNPA1 increased infiltration of T cells in vivo. (**A**) The images of the dissected tumors. (**B**) The growth curves of the tumors. (**C**)The weight of dissected tumors. (**D**) Immunohistochemistry analysis of CD4 and CD8 expression. Left: representative images, scale bar: 25 μm. Right: statistical graph. *n* = 5. Error bars, mean ± SD. (*P < 0.05, **P < 0.01, ***P < 0.001, ****P < 0.0001)

To further investigate the effect of HNRNPA1 knockdown on T cell activation in vivo, xenograft tumor models were established by subcutaneous injection of ID8 mouse ovarian cancer cells stably expressing shHNRNPA1 into C57BL/6J mice. Compared with the shNC group, downregulation of HNRNPA1 significantly inhibited ovarian cancer progression in vivo (Fig. 5A). Knockdown of HNRNPA1 slowed tumor growth and reduced tumor weight (Fig. 5B-C). The infiltration of CD4^+^ and CD8^+^ T cells in tumor tissues was then evaluated by immunohistochemistry. As shown in Fig. 5D, the level of CD8 positive signal was higher in HNRNPA1-sh1 group than that in shNC group, while no significant difference was observed in CD4 positive signal (Fig. 5D). It was demonstrated that downregulation of HNRNPA1 increased the infiltration of CD8^+^ T cells and inhibited the progression of ovarian cancer in vivo, suggesting the activation of CD8^+^ tumor-infiltrating T cells which thereby recognized and killed tumor cells.

## Discussion

Recognition of tumor-specific antigens is the key to tumor cell destruction in anti-tumor immunity (Matsushita et al. 2012; Tran et al. 2017). Lack or loss of tumor antigens is a major cause of immune escape and resistance to immunotherapy (Rosenthal et al. 2019; Anagnostou et al. 2017). High neoantigen levels are associated with more tumor-infiltrating T cells, improved response to immunotherapy, and better clinical outcomes (Accomando et al. 2020; Rizvi et al. 2015; McGranahan et al. 2016). In the present study, we found that disruption of the RNA-splicing factor, HNRNPA1, induced aberrant alternative splicing and generated neoantigens capable of priming T cell responses. It suggests that the induction of neoantigens via targeting RNA-splicing factor is a promising strategy to enhance anti-tumor immunity and improve immunotherapeutic efficacy.

Alternative splicing is a major source of neoantigens in cells. Novel mRNAs derived from alternative splicing events, including dysregulated exon skipping, aberrant inclusion of introns, exons with abnormal 3’ or 5’ ends, and etc., can potentially result in neoantigens. It has been reported that pharmacological disruption of the splicing factor RBM39 acutely induced tumor neoantigens and elicited anti-tumor immunity (Lu et al. 2021). Similar to this report, in the present study we also identified several neoantigens generated from transcript variants induced by HNRNPA1 downregulation. These results confirmed that disruption of AS in cells induced neoantigen generation. However, no concurrent ASEs were found in all 5 tumor cell lines included in our study, which differs from the finding that some ASEs were consistently found among cell lines (Lu et al. 2021). Only 1 SE and 1 A5SS were found to be consistently altered in SKOV3, HepG2, and K562. This may be due to the inherent character of tumor cells or to the differences in the data source. However, it is also possible that the ASE induced by HNRNPA1 downregulation varies from tumor to tumor. This may suggest that disruption of HNRNPA1, or any splicing factor, may induce different ASEs in different tumors or even individuals. Inclusion of more tumor types may provide more robust evidence. The immunogenicity of neoantigens translated from SKOV3-derived ASEs was calculated and further validated by IFN-γ ELISpots. We found that 11 out of 19 (∼ 58%) neoantigens could elicit T cells to secrete IFN-γ, which is higher than the ∼ 28% reported in the literature (Lu et al. 2021).Nowadays, several high-throughput sequencing technologies and bioinformatics pipelines help us to identify neoantigens and predict the immunogenicity of these candidates (Cheng et al. 2022). Few evidences have been reported to indicate which one is more accurate or efficient. We used both neoantigen prediction and immunity evaluation algorithms to obtain potential neoantigens and obtained a higher percentage of functional antigens. This suggests that a more thorough evaluation may be more efficient.

Currently, it is difficult to identify immunogenic neoantigens that are shared between patients (Bigot et al. 2021). And it is costly and complicated to identify personalized neoantigens for each patient, although it may achieve higher safety and efficacy. Identifying targets that can generate neoantigens with higher immunogenicity and abundance across patients may be a more economical and efficient strategy. Pan-cancer analysis showed that HNRNPA1 expression was elevated in many tumors (Fig. 1), indicating the potential role of HNRNPA1 in tumor therapy. In addition, HNRNPA1 expression levels are negatively correlated with the IPS score, which is considered to be a superior predictor of the response to immune checkpoint blockade (ICB), in a variety of malignancies (Charoentong et al. 2017). Personalized neoantigen vaccination combined with anti-PD-1 inhibitor could stimulate robust neoantigen-specific T-cell immunity and improve the efficacy of ICBs (Awad et al. 2022; Ott et al. 2020; Sun et al. 2023). In our study, we demonstrated that downregulation of HNRNPA1 could induce the generation of neoantigens that activate T cell responses. HNRNPA1 knockdown significantly increased CD8^+^ T cell infiltration and inhibited tumor progression in vivo. Furthermore, enrichment analysis revealed that several immune-related pathways, including cytokine- or chemokine-mediated pathways and antigen processing and presentation pathways, were enriched in HNRNPA1 knockdown cells, suggesting other potential mechanisms underlying the activation of anti-tumor immunity induced by HNRNPA1 knockdown aside from neoantigen induction. Taken together, these findings suggest that targeting HNRNPA1 in combination with anti-PD-1 therapy may be a potential immunotherapeutic regimen. Certainly, additional studies are needed to determine the feasibility and safety of this combination therapeutic strategy.

The present study had several limitations. The present study mainly used public transcriptomic data to analyze the correlation between HNRNPA1 expression and tumor immune microenvironment. Some confounding factors, such as interference from stromal cells, tumor cell heterogeneity, and unknown therapeutic history, may influence HNRNPA1 expression or alternative splicing patterns. Therefore, a more thorough investigation that may exclude the confounding factors will be more helpful. Additionally, the immunogenicity of neoantigens was only validated by ELISpot assay, and further in vitro and in vivo assays investigating neoantigen function, such as T cells proliferation, multiplex immunofluorescence staining and specific TCR T cells generation in pre-clinic models, should be performed in the future. Whether concurrent neoantigens should be induced by HNRNPA1 downregulation needs more thorough investigation, because few ASE were found among 5 tumor cell lines included for analysis in the present study. Neoantigens that bind to other MHC alleles should also be identified, which may also be applicable in tumor therapy.

## Conclusions

In summary, the present study found the negative role of HNRNPA1 in the TIME in many malignancies through pan-cancer analysis. Whole transcriptome analysis revealed that knockdown of HNRNPA1 could lead to aberrant alternative splicing events, which subsequently induce the generation of neoantigens. The IFN-γ ELISpot assay verified the immunogenicity of these candidate neoantigens in vitro. Downregulation of HNRNPA1 increased CD8^+^ T cell infiltration and inhibited tumor progression in vivo, suggesting that HNRNPA1 is a potential target for immunochemotherapy. These results have important implications for targeting the splicing factor, HNRNPA1, for cancer immunotherapy by inducing neoantigen production to elicit anti-tumor immunity. HNRNPA1 may serve as a novel target for tumor immunotherapy.

### Electronic supplementary material

Below is the link to the electronic supplementary material.

Supplementary Material 1

## Data Availability

The data from the TCGA, TARGET and GTEx that were analyzed in this study were obtained from the UCSC Xena at http://xena.ucsc.edu/. Expression profile data except SKOV3 were obtained from GEO at GSE88091, GSE80836, GSE87990, GSE80858, GSE71012, and GSE115654. The whole transcriptome sequencing data of SKOV3 are available upon request from the corresponding author.

## Abbreviations

A3SS: Alternative 3’splice site
A5SS: 5’Splice site
ACT: Adoptive cell therapy
AR: Androgen receptor
AS: Alternative splicing
ACC: Adrenocortical carcinoma
ALL: Acute lymphoblastic leukemia
ASE: Alternative splicing events
ATCC: American Type Culture Collection
BLCA: Bladder urothelial carcinoma
CESC: Endocervical adenocarcinoma
CHOL: Cholangiocarcinoma
CI: Confidence interval
COAD: Colon adenocarcinoma
CP: Immunomodulators/checkpoints
CTLA4: Cytotoxic T lymphocyte antigen-4
DEG: Differentially expressed genes
DSS: Disease-specific survival
EC: Effector cells
ELISpot: Enzyme-linked immunospots
GEO: Gene Expression Omnibus
GO: Gene Ontology
GTEx: Genotype-Tissue Expression
HGG: High-grade glioma
HNRNPA1: Heteronuclear ribonucleoprotein A1
HR: Hazard ratio
ICB: Immune checkpoint blockade
IEDB: Immune epitope database
ImmuCellAI: Immune Cell Abundance Identifier
KEGG: Kyoto Encyclopedia of Genes
KICH: Kidney chromophobe
KIRC: Kidney renal clear cell carcinoma
KIRP: Kidney renal papillary cell carcinoma
LAML: Acute myeloid leukemia
LGG: Brain lower-grade glioma
LIHC: Liver hepatocellular carcinoma
MSS: Microsatellite stability
MXE: Mutually exclusive exons
OS: Overall survival
OV: Ovarian serous cystadenocarcinoma
PBMC: Peripheral blood mononuclear cells
PCPG: pheochromocytoma and paraganglioma
PD-1: Programmed cell death protein 1
PK: Pyruvate kinase
PKM1: Pyruvate kinase M1 isoform
PKM2: Pyruvate kinase M2 isoform
RI: Retained intron
SARC: Sarcoma
SC: Immunosuppressive cells
SE: Skipped exon
SKCM: Skin cutaneous melanoma
TARGET: Therapeutically Applicable Research to Generate Effective Treatments
TCIA: Cancer Immune Group Atlas
TIME: Tumor immune microenvironment
UCEC: Uterine corpus endometrial carcinoma
UVM: Uveal melanoma
