# Supplementary material for: Downregulation of HNRNPA1 induced neoantigen generation via regulating alternative splicing
Source: Mol Med. 2024 Jun 12;30:85. doi: 10.1186/s10020-024-00849-0 (PMC11167825; doi:10.1186/s10020-024-00849-0)
Supplement: Supplementary file 1 — Supplementary Material 1 [file 10020_2024_849_MOESM1_ESM.pdf]

Supplementary Table S1: The detailed information of 4 GEO datasets.

GEO	Samples	GPL	Citation
GSE88091	shHNRNPA1 HepG2 (n=2)	Illumina HiSeq 2000	[1]
GSE80836	shHNC HepG2 (n=2)	Illumina HiSeq 2000	
GSE87990	shHNRNPA1 K562 (n=2)	Illumina HiSeq 2000	
GSE80858	shNC K562 (n=2)	Illumina HiSeq 2000	
GSE71012	siNC MCF-7M (n=2)	Illumina HiSeq 2000	[2]
	siHNRNPA1 MCF-7M (n=2)		
GSE115654	Ctrl B-LL (n=2)	Illumina HiSeq 2500	[3]
	HNRNPA1-KD B-LL(n=2)		

[1] An integrated encyclopedia of DNA elements in the human genome. Nature, 2012. 489(7414): p. 57-74.

[2] Yang, J., B.D. Bennett, S. Luo, et al., LIN28A Modulates Splicing and Gene Expression Programs in Breast Cancer Cells. Molecular and Cellular Biology, 2015. 35(18): p. 3225-3243.

[3] Black, K.L., A.S. Naqvi, M. Asnani, et al., Aberrant splicing in B-cell acute lymphoblastic leukemia. Nucleic Acids Research, 2018. 46(21): p. 11357-11369.

Supplementary Table S2: Sequences of primers for qRT-PCR.

Name	Sequence
HNRNPA1-F	GGCCTTGCATTCATAGCTGC
HNRNPA1-R	GCTCACGGACTGTGTGGTAA
ABCC3-F	GGTTCACAAAGGCTGCTAAACC
ABCC3-R	ATGGCCATCCTGGGGGGAGGT
ADGRL3-F	GTTAAAGATATCTCTACCACAGG
ADGRL3-R	CAAGAGGTCCCTATAGTTCCTG
CERKL-F	CATTGGTAGATCTTGTTACATCAG
CERKL-R	TATTGGCAGGCTTTCCAAACAG
CIT-F	CCAAGCAGTTTCTGAACCCAGGC
CIT-R	GAAGCTGATGCTGCCCGCGAC
FBXO30-F	AGTCCAGCTCGGCCGGCGCG
FBXO30-R	TGTGGGGTACGGAGCGTC
HPS1-F	GCACTGTGGCTGACAGCTTC
HPS1-R	TCGCCGTGGAGGTGACTAC
KIT-F	GCAGCGATAGTACTAATGAG
KIT-R	CTATTCTCACAGATCTCCTT
MAP2-F	CTAAAGGGGGGCAGGTTAGGATT
MAP2-R	GGTAACAATTTGTACATTTCCGCC
POFUT2-F	CCTGCCATGCTTTAGAGCCAG
POFUT2-R	GTTGCCACAGGGATGGAGG
POLN-F	GATCTTGTGAACCTGCCTGTAT
POLN-R	CAGAAGCAGTGTTAAATGCTC

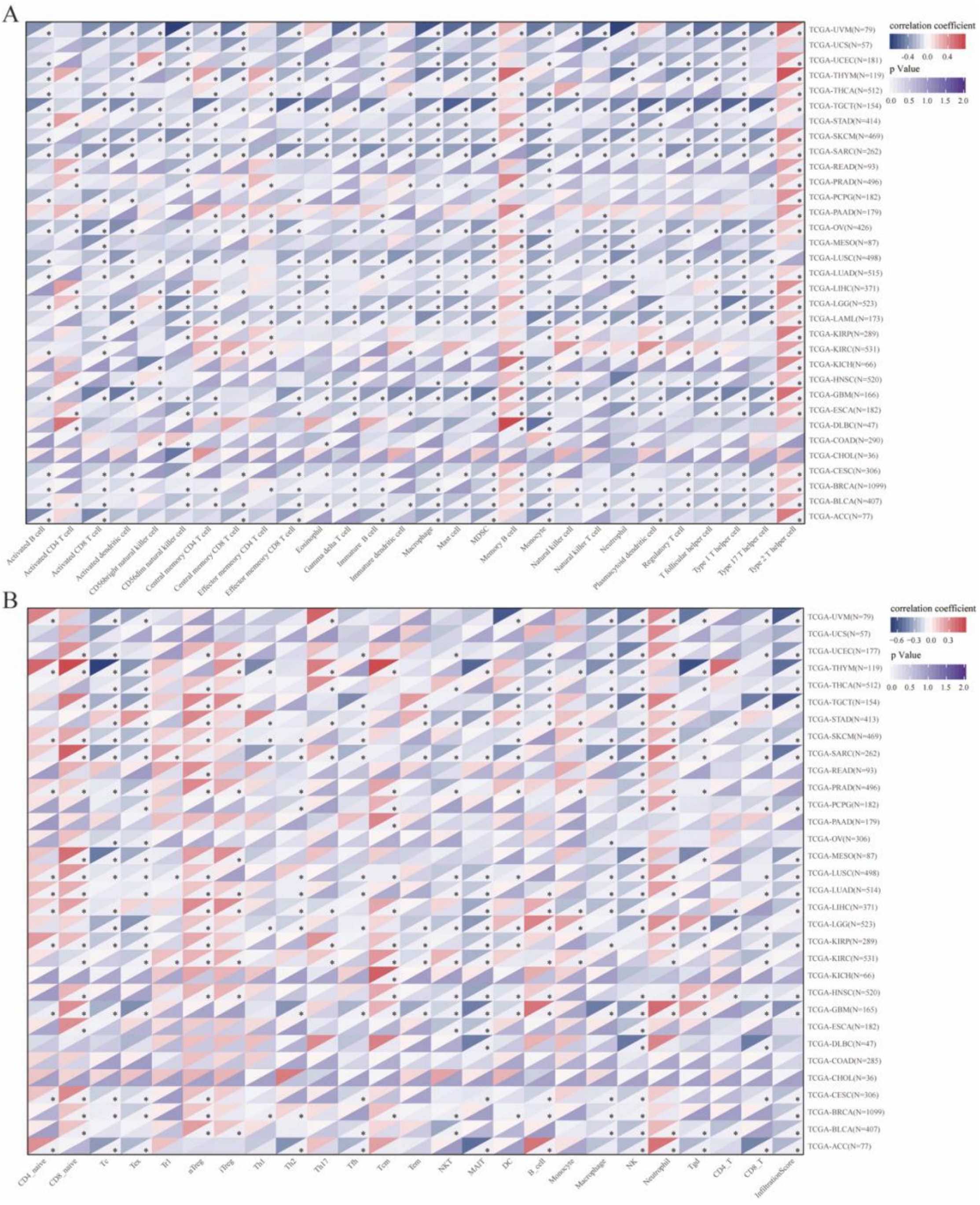

Supplementary Fig.1 Correlation of HNRNPA1 expression with immune cell infiltration. (A) ssGSEA. (B) ImmuCellAI.

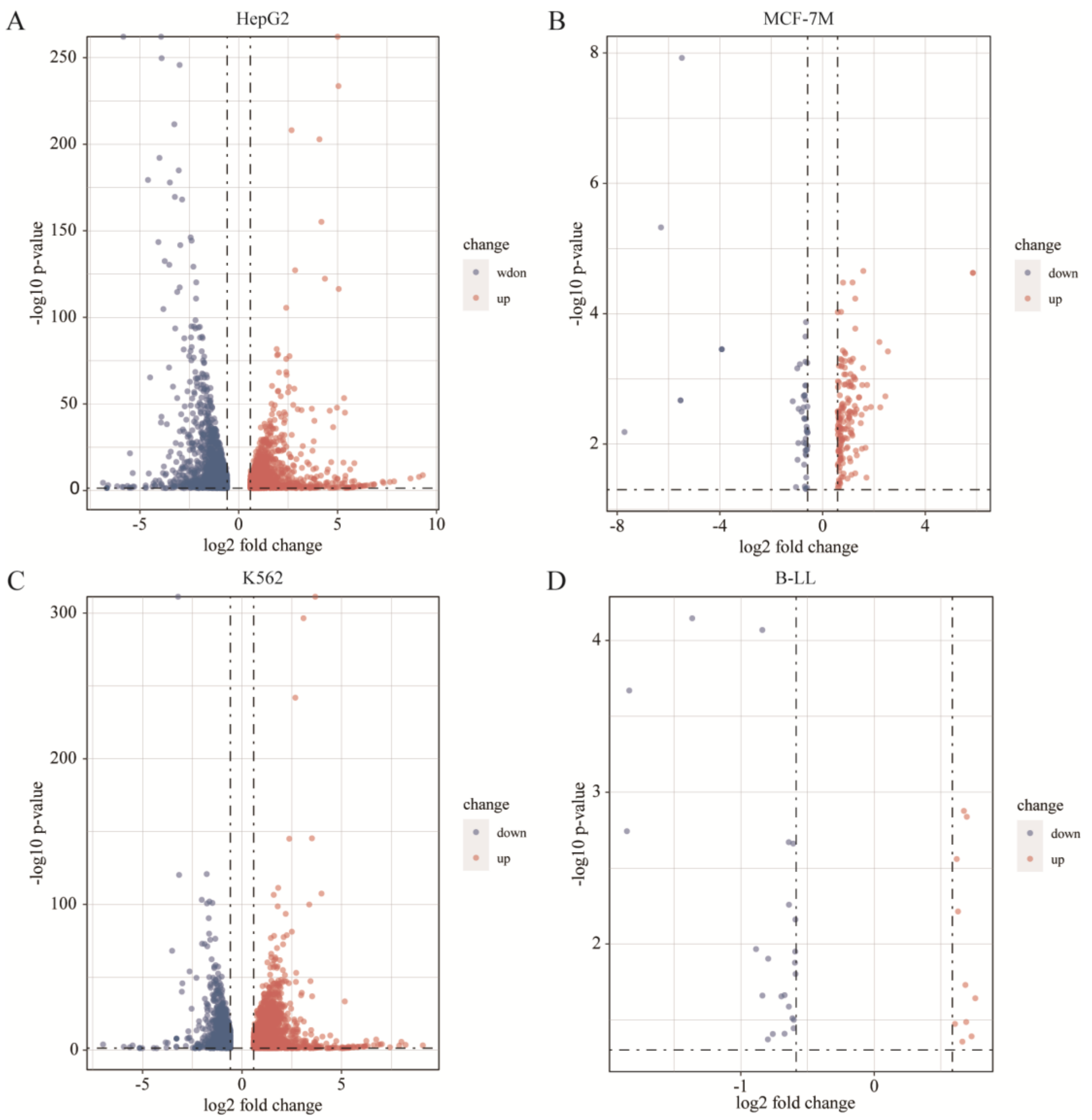

Supplementary Fig.2 Identification of DEGs in shHNRNPA1 cells. The volcano plot of the differentially expressed genes in shHNRNPA1 cells**.** The threshold is fold change> 1.5 and *P value* < 0.05. The up-regulated genes are shown in red, while the down-regulated genes are shown in blue. (A) HepG2. (B) MCF-7M. (C) K562. (D) B-LL.

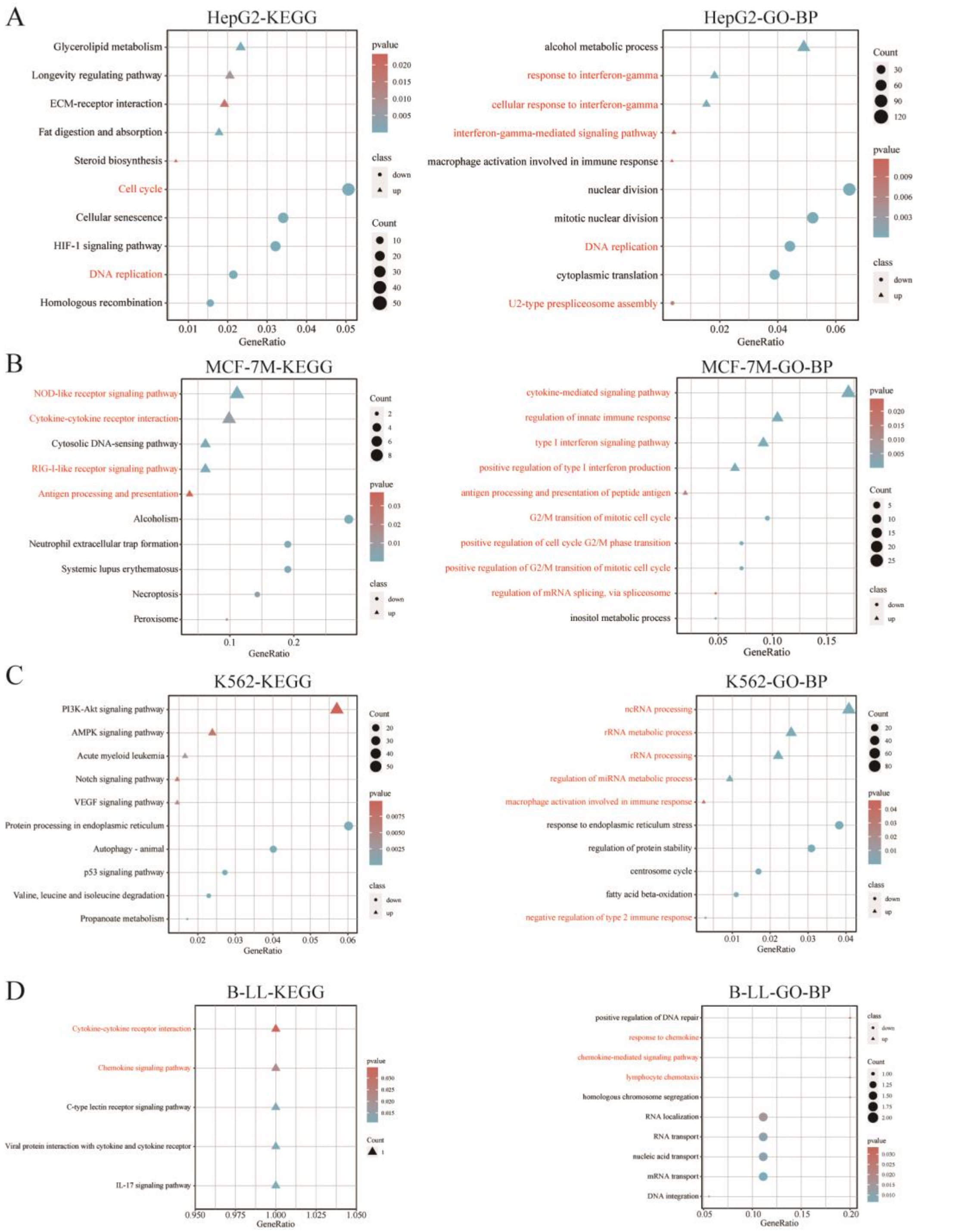

Supplementary Fig.3 Functional enrichment analysis of DEGs in shHNRNPA1 cells. Enriched KEGG and GO terms based on DEGs. Threshold is *P value* < 0.05. (A) HepG2. (B) MCF-7M. (C) K562. (D) B-LL.

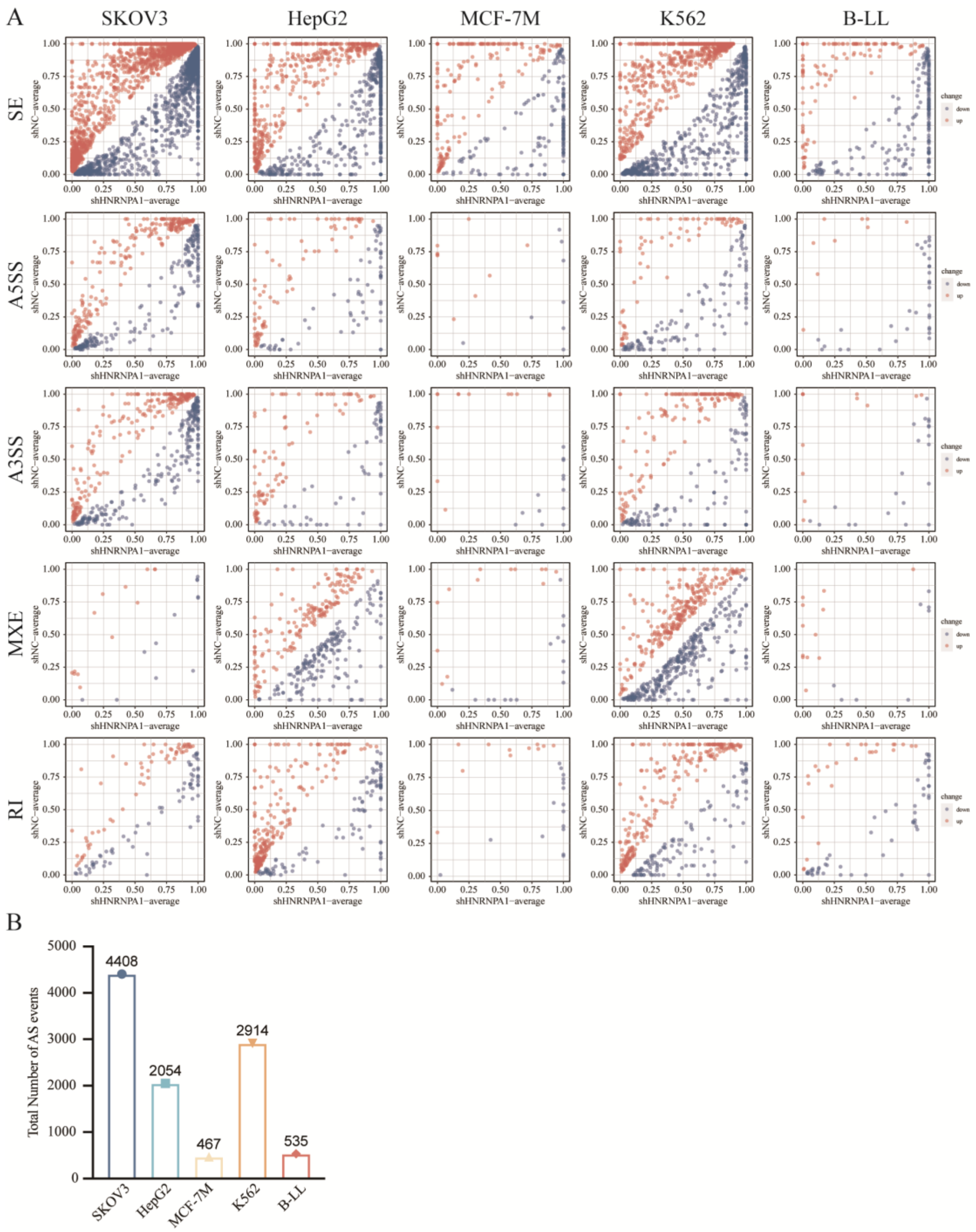

Supplementary Fig.4 The dysregulated alternative splicing events induced by HNRNPA1 downregulation. (A) The dot plot of five basic types of AS events in five tumor cell lines. The threshold is FDR <= 0.05 and |IncLevelDifference|>0.01. The up-regulated AS are shown in red, while the down-regulated AS are shown in blue. (B) The total number of dysregulated alternative splicing events induced by HNRNPA1 downregulation in five tumor cell lines.

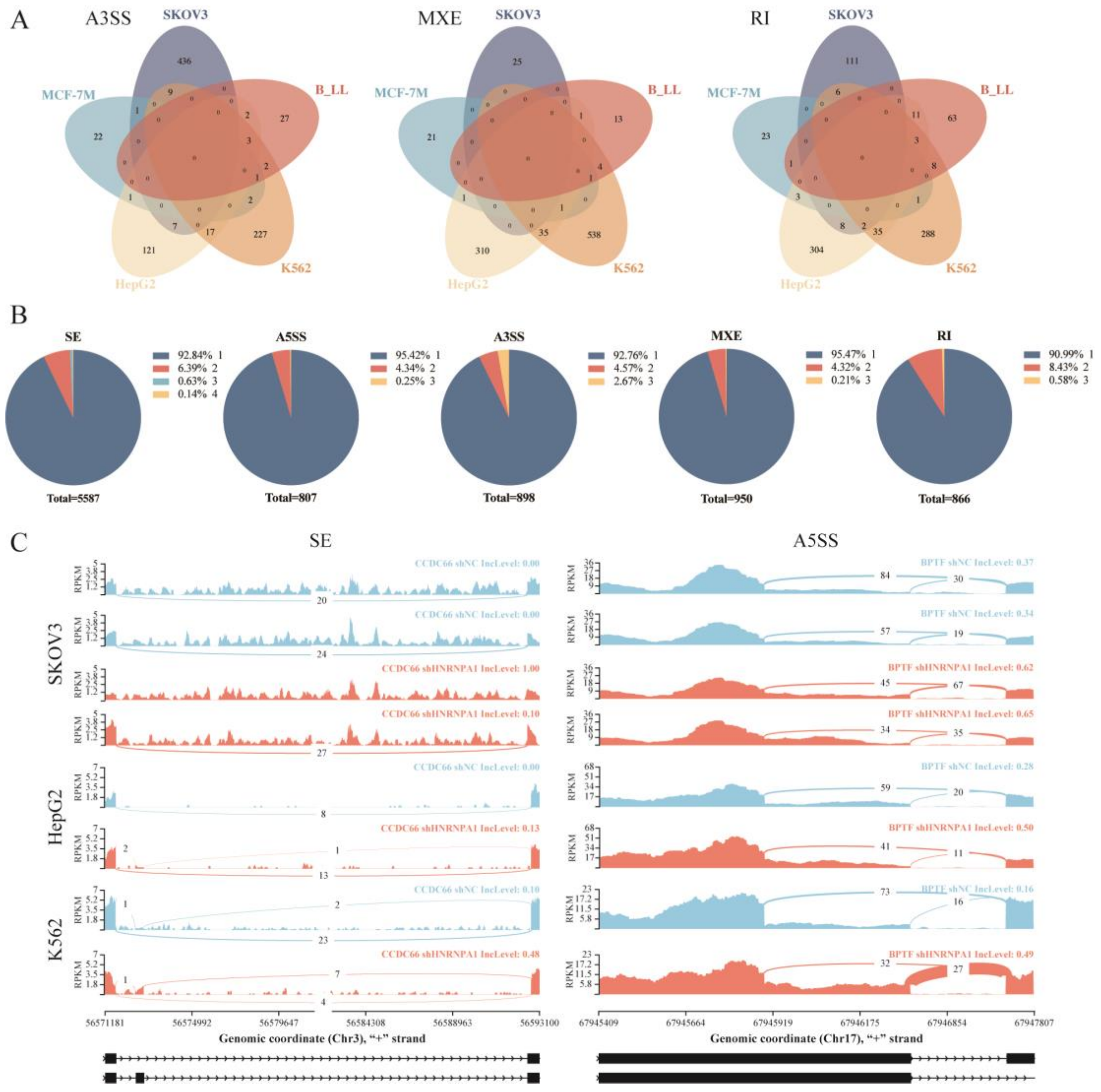

Supplementary Fig.5 Consistent alternative splicing events shared by tumor cells. (A) The Venn diagram of alternative splicing events, A3SS, MXE, and RI, shared by five tumor cell lines. (B) The percentage of consistent alternative splicing events. (C)Sashimi plot illustrating shared skipped exon (left) and 5'splice site (right) induced by HNRNPA1 downregulation in SKOV3, HepG2, and K562.

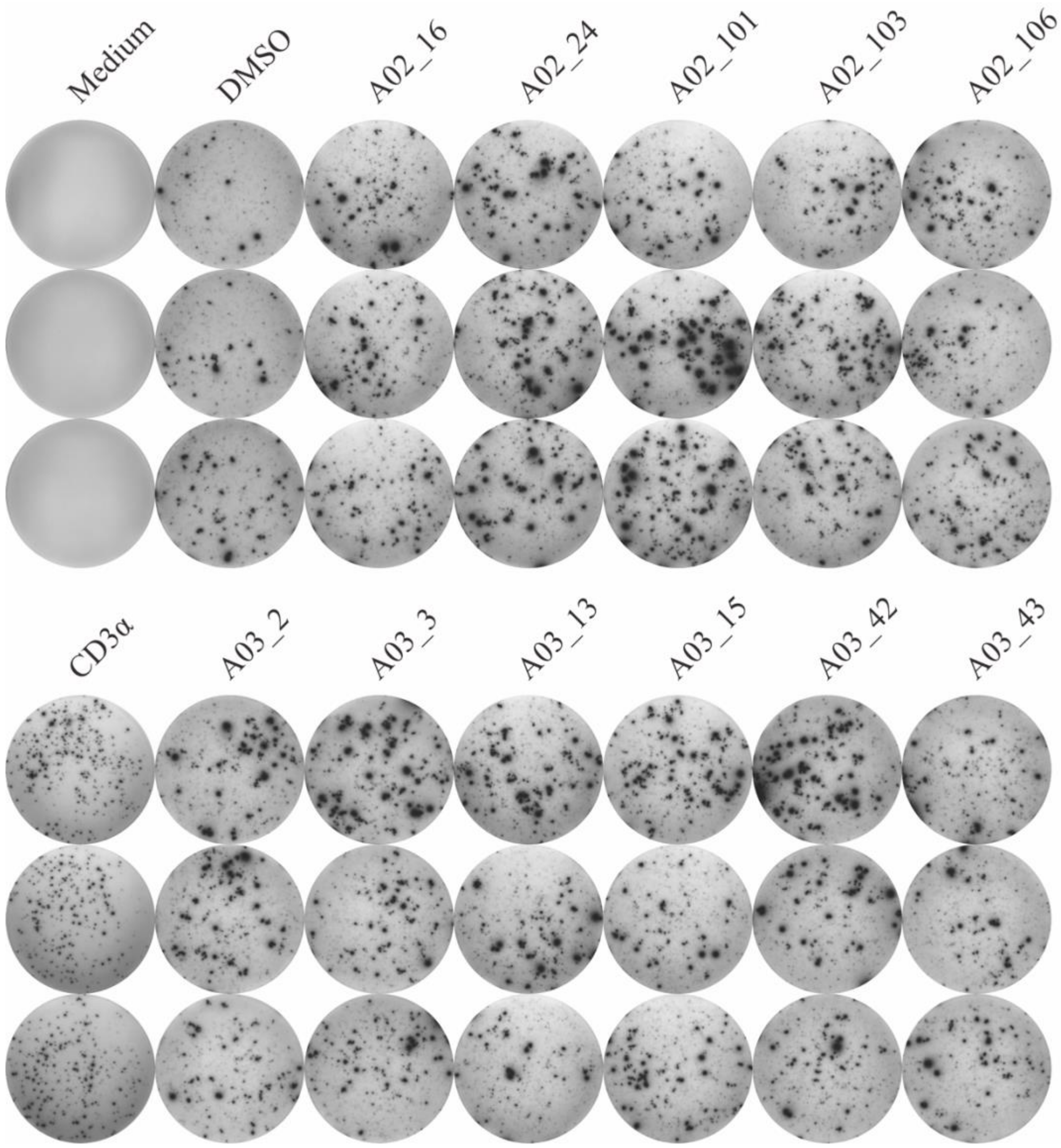

Supplementary Fig.6 Illustration of medium, DMSO, CD3α, and neoantigens IFN-γ ELISpots wells.
